# Localized Breakdown of the Niobium Anodic Oxide Film by Bromides

**DOI:** 10.1002/chem.202500398

**Published:** 2025-05-03

**Authors:** Eirini Lappa, Kyriaki Saltidou, Chrysanthi Gkili, Dimitra Sazou

**Affiliations:** ^1^ Department of Chemistry Aristotle University of Thessaloniki, University Campus Thessaloniki 54124 Greece

**Keywords:** bromides, localized oxide breakdown, niobium anodic oxide, point defect model, vacancy condensation references

## Abstract

The growth of the anodic oxide passive film on Nb and its stability were studied in aqueous bromide media. Potentiodynamic polarization up to 12 V_SCE_ showed that passive Nb loses its stability resulting in either metastable or stable pitting corrosion depending on the bromide concentration and the potential scan rate. On the contrary, Nb remains passive in the more aggressive chloride media where most passive metals and alloys are often susceptible to localized breakdown. This unusual bromide‐induced localized breakdown of passive Nb occurs for bromide concentrations higher than 0.25 M at a critical breakdown potential (*E*_b_). Stable active dissolution in pits was observed at potentials higher than *E*_b_. Το get an insight into the physicochemical processes responsible for the susceptibility of Nb to the bromide‐induced localized corrosion, the oxide growth and its breakdown are discussed in terms of a point defect model (PDM). Experimental relationships between *E*_b_ and either the bromide activity or the potential scan rate agree with the PDM predictions. The bromide‐induced localized breakdown of passive Nb seems to be associated with the absorption of bromides preferably on lattice anion vacancies at the oxide|electrolyte interface leading through a series of processes to vacancy condensation at the Nb|oxide interface.

## Graphical abstract

The bromide‐induced localized breakdown of niobium anodic oxide films depends on the bromide concentration and potential scan rate. Related experimental relationships agree with the predictions of a point defect model (PDM) under certain conditions. Within the same potential region, niobium maintains passivity in the more aggressive chloride media. PDM is used to explain the bromide‐induced localized corrosion of niobium

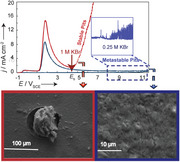

## 1 Introduction

Niobium belongs to group V valve metals that have the tendency to form passive oxide films on their surface.^[^ ^1^ ^]^ The transition of Nb to its passive state is facilitated under ambient conditions in air and in aqueous solutions due to its great affinity to oxygen. A “native” oxide film of 1–3 nm in thickness exists on Nb surface separating metal substrate from the environment.^[^ ^2^ ^]^ Therefore, niobium has been used in the past in the industry of metallurgy to replace tungsten in steel tools and to stabilize stainless steel against surface corrosion. In recent years, applications of niobium and its porous oxide films have further been extended towards different directions such as medical implants, superconductors, batteries, supercapacitors, gas sensors, and memristive devices.^[^ ^3^ ^]^

The kinetics and mechanism of the oxide growth on Nb and other valve metals were investigated under anodic polarization conditions in different aqueous electrolytic media.^[^ ^4^ ^]^ The electrolyte composition and electrochemical parameters found to play a decisive role on the growth of the anodic oxide on Nb and hence on its performance in possible applications.^[^ ^3e,f^ ^]^ The oxide film grows to a thickness approximately proportional to the formation potential imposed across the Nb|electrolyte interface. Upon scanning the potential from the equilibrium state of the Nb|electrolyte interface towards the anodic region, the transition of Nb to its passive state can be detected in the current‐potential curve. The active‐passive transition of Nb occurs by a low corrosion rate in most acidic media of various concentrations and temperatures.^[^ ^5^ ^]^ Investigations of the Nb electrochemical behavior in concentrated sulfuric acid solutions (20, 40, 80 wt%) and hydrochloric acid solutions (20, 38 wt%) at temperatures of 25, 75, and 95 °C have shown that Nb remained in its passive state and the corrosion rate did not exceed 306 µm/yr.^[^ ^6^ ^]^ At high temperatures, the corrosion rate of Nb increased causing general corrosion through the chemical dissolution of Nb_2_O_5_. General corrosion of Nb at higher rates occurs also in concentrated alkaline solutions.^[^ ^7^ ^]^ At concentrations greater than 10 wt% NaOH and temperatures above 25 °C the passive oxide dissolves and the surface is activated forming soluble NbO_3_^−^ whereas the corrosion rate increases by increasing the concentration of NaOH.

Moreover, dissolution of oxide films on Nb and other valve metals occurs in acidic fluoride solutions resulting in electropolishing for high‐field superconducting radiofrequency cavity applications.^[^ ^8^ ^]^ As fluoride‐containing media are mainly used to obtain nanostructures on Nb and other valve metals, several models have been suggested to explain the oxide growth‐dissolution process resulting in the formation of the Nb nanoporous oxide.^[^ ^9^ ^]^ The effect of other halides on the stability of the passivity on Nb is not clarified yet. It is well‐known that halides (X^−^ ≡ Cl^−^, Br^−^, I^−^) may induce local breakdown of the metal passive surfaces resulting in pitting corrosion and that the halide aggressiveness varies in the order Cl^−^ > Br^−^ > I^−^. However, in the case of valve metals this order seems invalid as under certain conditions, Cl^−^ found to be less aggressive than Br^−^.

For example, the oxide on Nb grown under potentiostatic conditions at various potentials in HCl solutions found to be stable.^[^ ^10^ ^]^ Similarly, Nb seems to retain passivity at ambient temperatures in bromine‐containing HBr solutions due to the presence of bromine.^[^ ^11^ ^]^ On the other hand, other studies have shown that after the potentiodynamic polarization of pre‐passivated Nb surfaces in 3.5 wt% NaCl, tiny and deep pits appear on the surface, when the pre‐passivation was performed in H_2_SO_4_ and HCl solutions, respectively, but anodic breakdown of the passivity on Nb due to the chloride attack is not reported.^[^ ^12^ ^]^ On the contrary, passive films of Nb exhibit a high susceptibility to localized breakdown by bromides.^[^ ^13^ ^]^ Radioactive tracer measurements detected bromide adsorption when the oxide film starts to thicken. This along with local measurements of the potential led to the conclusion that anodic oxide film has defect sites with an increased electron conductivity where the breakdown may occur.^[^ ^13b^ ^]^ Such spatial electrochemical activity was also found for the Ti|TiO_2_ interface by using scanning electrochemical microscopy in bromide‐containing solutions where bromine generation occurred.^[^ ^14^ ^]^ The ability of titanium to maintain passive film in chloride solutions but not in bromide is known for a long time.^[^ ^15^ ^]^ This behavior was further explored and a rational for the unusual susceptibility of Ti to breakdown by the attack of Br^−^ was proposed within the context of a point defect model (PDM).^[^ ^16^ ^]^

The PDM was suggested to describe the anodic oxide growth and its breakdown on metals and alloys considering the presence and distribution of point defects (cation and anion vacancies and metal interstitials) across the metal|oxide and oxide|electrolyte interfaces along with the interfacial potential drop, electrolyte composition, and temperature.^[^ ^17^ ^]^ Though the PDM and its variants have effectively been utilized to understand the kinetic of the anodic oxide growth on Nb it was not yet exploited to describe the localized passivity breakdown on Nb by halides and explain the greater aggressiveness of bromides as compared with that of chlorides.

In the present work, the transition of Nb from the passive state to the localized oxide breakdown state and subsequent pit active dissolution is investigated in KBr aqueous solutions of different concentrations using anodic potentiodynamic polarization up to 12 V_SCE_ to define the critical breakdown potential. The main objective of this investigation is to characterize systematically the higher susceptibility of passive oxide films on Nb to the bromide‐induced localized corrosion and try to get a physical insight to this effect, which has not been investigated previously. The critical breakdown potential of the passive state is defined as a function of the bromide activity and the potential scan rate whereas the bromide effect on the electronic properties of the passive film is evaluated using the Mott‐Schottky analysis. The oxide growth and its breakdown are discussed within the context of a PDM.

## 2 Results

### 2.1 Potentiodynamic Polarization of Nb in Various Media

**Figure 1:**
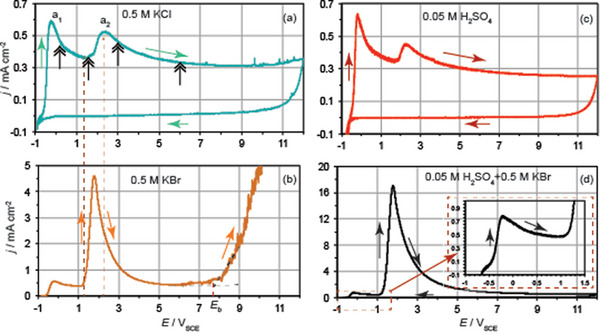
Current density‐potential (*j*‐*E*) potentiodynamic curves of Nb traced at *υ* = 50 mV s^−1^ within the region −0.9 V – 12 V in (a) 0.5 M KCl, (b) 0.5 M KBr, (c) 0.05 M H_2_SO_4_, and (d) 0.05 M H_2_SO_4_+0.5 M KBr. Arrows indicate the direction of the potential scan. The inset in panel (d) depicts a magnification of the transition to the passive state at peak **a_1_**.

Figure 1 illustrates the current density‐potential (*j*–*E*) polarization curves of Nb traced in 0.5 M KX (X^−^ ≡ Cl^−^, Br^−^) solutions at a potential scan rate, *υ* = 50 mV s^−1^ within the potential region −0.9 V − 12 V in comparison with the corresponding *j*–*E* curves in 0.05 M H_2_SO_4_ in the absence and presence of 0.5 M KBr. The open circuit potential, *E*_OC_ of the Nb|Nb_2_O_5_ |electrolyte interfacial system measured immediately after the immersion of the Nb electrode in the neutral halide media was found to be slightly lower than the *E*_OC_ measured in acidic media. After 30 s, the *E*_OC_ is close to ∼ −0.4 V_SCE_ in the former case and to −0.35 V_SCE_ in the latter one. Significant variations were not detected for the *E*_OC_ upon varying either the nature of the halide or the concentration of the KX solutions under study. The measured *E*_OC_ values indicate that a thin native oxide film of almost same thickness exists on the Nb surface immersed in various KX solutions, depending only on the electrode pre‐treatment.

During the forward potential scan at *υ* = 50 mV s^−1^ within the region between −0.9 and 12 V, a peak **a_1_** appears with a peak potential, *E*_p__a1_ ∼ −0.25 – −0.23 V in all media used in this study. Peak **a_1_** signifies the formation of an anodic oxide film on Nb associated with its transition to the passive state according to the overall reaction:

(1) $$2\mathrm{Nb}+5H_{2}O\;\to Nb_{2}O_{5}+10H^{+}+10e^{-}$$

Moreover, further oxidation of the oxide at the areas where the metal is not in its highest oxidation state may occur in this potential region.^[^ ^13a^ ^]^ Beyond the *E*_p__a1_, the anodic current reaches a plateau within a limited potential region and in turn increases up to peak **a_2_** at *E*_p__a2_ ∼ 2.3 V in bromide‐free solutions (Figures 1a and c). This corresponds to the beginning of the oxygen evolution reaction (OER) at localized electroactive sites. Though the OER is assumed to prevail in both sulphate and chloride solutions ^[^ ^3b^ ^]^, the chlorine evolution reaction (CER) (*E* ^0^ = 1.6 V_SCE_) could not be excluded to occur from chloride solutions. If the CER would occur, the corresponding peak of the chloride electrochemical oxidation would be overlapped with that of the OER. Inspecting comparatively the *j–E* curves of Figure S1 shows that the **a_2_** peak current density, *j*_p,a2_ increases only slightly in 0.5 M KCl as compared with that in sulfuric acid solutions where the *j*_p,a2_ is mainly assigned to OER. Additionally, upon increasing the chloride concentration to 1 M, the *j*_p,a2_ does not increase remarkably as can be seen in the *j‐E* curve traced in 1 M KCl (Figure S2). These observations show that the CER occurs to a rather limited extent. Indeed, the *j*_p,a2_ is approximately ∼25 times lower in 0.5 M KCl than in 0.5 M KBr (Figure 1b) where the electrochemical oxidation of bromides (*E* ^0^ = 1.33 V_SCE_) does occur. Upon increasing the potential beyond the *E*_p__a2_, the thickness of the oxide film increases resulting in a lower conductivity and hence a lower current density.^[^ ^18^ ^]^ During the reverse backward potential scan, the current remains low within the whole potential region due to the existence of a passivating oxide layer, a typical behaviour of valve (rectifying) metals.

In the case of 0.5 M KBr (Figure 1b) and bromide‐containing 0.05 M H_2_SO_4_ (Figure 1d), overlapped with the peak **a_2_** due to OER, there exist an additional anodic peak around 2 V, which is attributed to the bromide oxidation to bromine. Similar behavior was also observed in the case of Ti in bromide solutions where under appropriate conditions, bromides induced a localized breakdown of the oxide film at higher potential values.^[^ ^14^, ^16^ ^]^ As can be seen by the pronounced increase of the current beyond a critical breakdown potential, *E*_b_, this is also the case for Nb in 0.5 M KBr (Figure 1b). Localized breakdown of the oxide film and in turn, a passive‐active transition is prevented in the presence of other anions such as sulfates (Figure 1d). Interestingly, the **a2** peak current density increases remarkably in the presence of sulfates indicating a catalytic effect of the sulfate‐modified surface of the niobium oxide^[^ ^19^ ^]^ for the bromide oxidation. The catalytic effect is enhanced in 0.05 M H_2_SO_4_ as compared with the 0.2 M Na_2_SO_4_ solution (Figure S3) and cannot be attributed to the increase of the solution conductivity.^[^ ^20^ ^]^ However, beyond the *E*p_a2_, sulfates and bromides both compete for adsorption sites on the oxide inhibiting the oxide breakdown as seen by the shift of the *E*_b_ towards higher values.^[^ ^14^, ^16^ ^]^ Oxygen vacancies can be considered as the sites at which bromides could be adsorbed to induce the localized passivity breakdown on Nb, as will be discussed below.

### 2.2 Growth of the Anodic Oxide Film on Nb

**Scheme 1:**
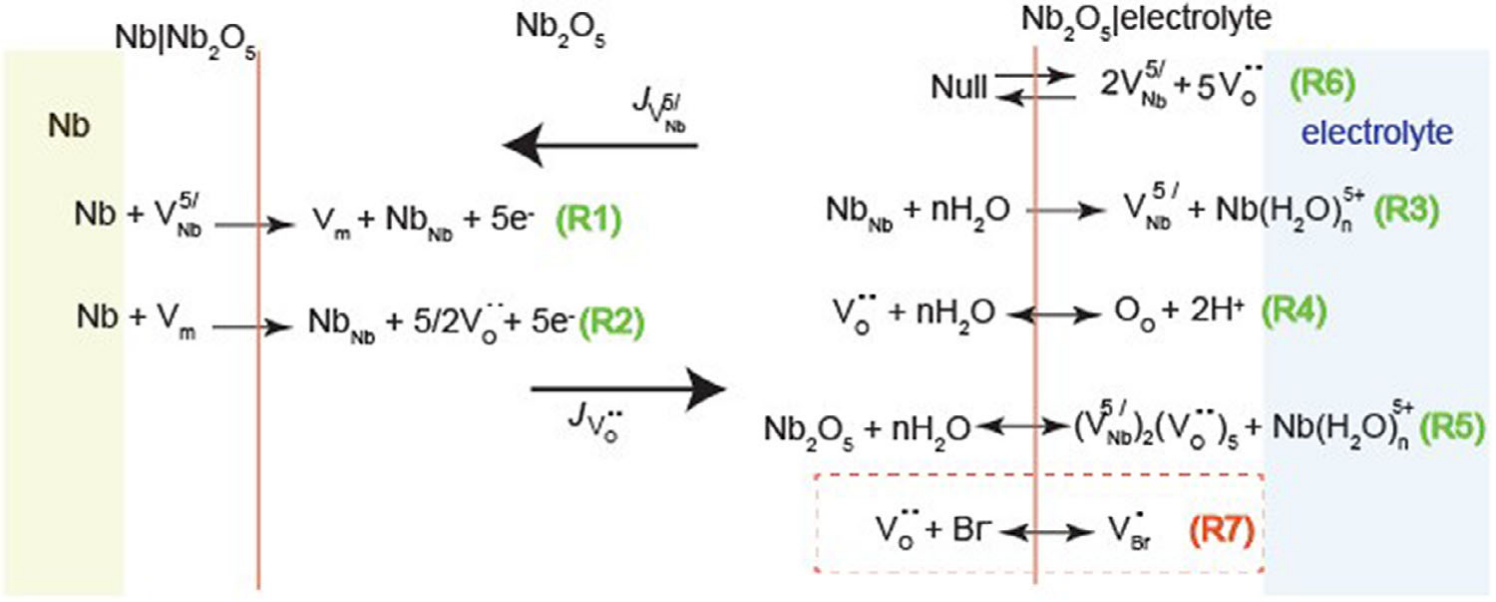
Illustration of reactions occurring across the Nb|Nb_2_O_5_ and Nb_2_O_5_|solution interfaces and transport processes by which oxygen vacancies, $V_{\ddot{O}}$ and cation vacancies, $V_{\mathrm{Nb}}^{5^{\prime }}$ are transferred through the thin oxide film. Fluxes $J_{V_{\mathrm{Nb}}^{5^{\prime }}}$, $\;J_{V_{\ddot{O}}}$ of cation and anion vacancies, respectively, are indicated by arrows. The Kroger‐Vink notation has been used. The «V» denotes a vacancy, subscripts denote vacant positions in the oxide lattice, and superscripts denote positive «′» and negative «**^⋅^** » effective charges.

The point defect model (PDM) proposed first by Macdonald describes in atomic scale the generation and annihilation of point defects during the growth of the anodic oxide film on metals.^[^ ^17^ ^]^ It provides an adequate explanation for the metal passivity under steady‐state conditions as well as its local breakdown in the case of several metals and alloys.^[^ ^21^ ^]^ For the anodization of Nb, variants of the PDM have also been used for the quantitative characterization of passive oxide films in alkaline media ^[^ ^22^ ^]^ and in fluoride‐containing sulfate solutions.^[^ ^23^ ^]^ According to the PDM the oxide defective structure comprises metal and oxygen vacancies and perhaps metal interstitials, the formation of which, however, is considered less favorable during Nb passivation. Scheme 1 summarizes basic reactions assumed to occur across the Nb|Nb_2_O_5_ and Nb_2_O_5_|electrolyte interfaces along with transport processes taking place within the passive film under the influence of an electric film. Reaction (R1) refers to the submergence of niobium vacancies, $V_{\mathrm{Nb}}^{5^{\prime }}$ into the Nb lattice and the generation of Nb_Nb_ in the oxide lattice while a vacancy *V*_m_ remains in the metal lattice. The vacancy *V*_m_ is involved in reaction (R2), which leads to the generation of oxygen vacancies, $V_{\ddot{O}}$ indicating a shift of the Nb|Nb_2_O_5_ surface towards the metal side. The metal vacancies $V_{\mathrm{Nb}}^{5^{\prime }}$ are generated across the Nb_2_O_5_|electrolyte interface via the reaction (R3) and transferred by the flux $J_{V_{\mathrm{Nb}}^{5^{\prime }}}$ to the Nb|Nb_2_O_5_ interface where they are annihilated via reaction (R1) as can be seen in Scheme 1. On the other hand, oxygen vacancies, $V_{\ddot{O}}$ generated at the Nb|Nb_2_O_5_ interface are transferred via the flux $J_{V_{\ddot{O}}}$ to the Nb_2_O_5_|electrolyte interface where they are occupied by O^2−^ from H_2_O according to reaction (R4). Reaction (R4) results in the lattice oxygen O_O_ that are transferred across the oxide film via a hopping mechanism towards the opposite direction with respect to the flux $\;J_{V_{\ddot{O}}}$.

Reaction (R5) represents the dissolution of the passive oxide film, which though occurs at a relatively low rate in the case of valve metals, it is required for steady state conditions of the passivity on Nb to be considered. The equilibrium (R6) denotes Schottky disorder according to which an electrically neutral anion/cation pair leaves normal lattice sites for new surface sites whereas a neutral vacancy pair stays in the bulk oxide. It is important to note that electrolyte anions may also occupy oxygen vacancies at the Nb_2_O_5_|electrolyte interface as reaction (R7) shows in the case of bromide‐containing solutions.^[^ ^16^, ^24^ ^]^ The latter reaction will further be discussed below as it is related to the perturbation of the Schottky equilibrium and a bromide‐catalyzed generation of cation/anion pair vacancies that may initiate the localized breakdown of the passivity on Nb.

### 2.3 Mott‐Schottky Analysis

**Figure 2:**
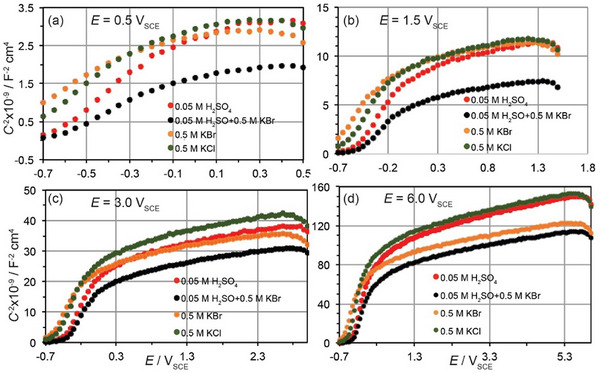
Mott‐Schottky plots ($C^{-2}$ versus *E*) for Nb oxide films formed in the same media from which the *j*‐*E* curves shown in Figure 1 were obtained. For the M–S plots the potential was scanned at *υ* = 10 mV s^−1^ from −0.9 V up to the (a) 0.5 V, (b) 1.5 V, (c) 3 V, and (d) 6 V as the double arrows indicate in Figure 1a.

Mott‐Schottky (M–S) analysis was performed to study the effect of the electrolyte composition on the electronic properties of the anodic oxide film formed on Nb and evaluate the donor density, *N*_D_. Εvaluated *N*_D_ values are indicative of changes caused by different anions under specific conditions. M–S curves were traced at selected potential values in bromide‐containing solutions in comparison with other media. The selected potentials include *E* = 0.5 V where the oxide has already been formed causing passivation, *E* = 1.5 V where either OER or bromide oxidation may occur on the passive Nb resulting in a decrease of the oxide resistance and more positive potentials, *E* = 3 and 6 V, where the resistance of the oxide film starts to increase again due to the increase of the oxide thickness. Figure 2 shows comparative M–S plots ($C^{-2}$ versus *E*) of the passive Nb in the same solutions which were used to trace the voltammograms of Figure 1, where *C* is the capacity of the space charge layer. The slope of the $C^{-2}$ – *E* plots over the passive range between 0.5 and 6 V is positive indicating that the niobium oxide film behaves as an *n*‐type semiconductor corroborating previous studies in other media.^[^ ^10^, ^25^ ^]^ Anion vacancies or cation interstitials may be the dominant defects and certainly M–S analysis cannot differentiate between them. However, due to the lower formation energy of oxygen vacancies as compared with the formation energy of niobium interstitials, the former is considered to prevail in the barrier layer.

The M–S curves at *E* > 1.5 V display nonlinearity and for media of the same pH they converge approximately on the flat band potential, *E*_FB_, which is ranged between −0.7 and −0.5 V_SCE_. The bending of M–S curves has also been observed for other valve metals in various media. It has been assigned to a number of factors including nonuniform distribution of donor states in the oxide film, surface roughness, the presence of surface states changing the potential drop across the double layer, distribution of donor states over an extensive range of energies or the presence of a second donor level in the band gap that is not ionized at *E*_FB_.^[^ ^26^ ^]^ The donor density can be evaluated based on the M–S equation,

(2) $$\;\frac{1}{C^{2}}=\frac{2}{\varepsilon \varepsilon_{o}eN_{D}A^{2}}\left(E-E_{\mathrm{FB}}-\frac{\mathrm{kT}}{e}\right)$$

where *ε* is the dielectric constant of the oxide (*ε* = 41.4)^[^ ^27^ ^]^ *ε*_o_ is the vacuum permittivity, *e* is the charge of the electron (*e* = 1.602×10^−19^ C), *N*_D_ is the donor density, *A* is the geometrical surface area, *E* and *E*_FB_ is the applied potential and flat band potential, respectively, *k* is Boltzmann's constant (1.38×10^−23^ J K^−1^) and *T* is the ambient temperature (∼293 K). According to Equation (2), *N*_D_ values were calculated from the slope of the linear part of the M–S plots at applied potentials used in different media and they are summarized in Table 1.

**Table 1:** Estimated donor density, *N*_D_ values of the anodic oxide film formed on Nb at different applied potentials.

*N*_D_x10^−20^/cm^−3^				
*E/V*_SCE_	KCl 0.5 M	KBr 0.5 M^[^ ^a^ ^]^	H_2_SO_4_ 0.05 M	H_2_SO_4_+KBr (0.05+0.5 M)
0.5	11.91	16.28	8.66	13.52
1.5	2.65	3.98	2.32	3.50
3.0	0.73	1.00	0.77	0.99
6.0	0.24	0.44	0.22	0.29

^[a]^ Further increase of bromide concentration results in increasing *N*_D_.

The donor density increases in bromide‐containing media. Lower values of *N*_D_ were found in chloride‐containing media of the same concentration compared to bromide‐containing media. Even lower is the *N*_D_ in 0.05 M H_2_SO_4_. The *N*_D_ decreases upon increasing the potential in agreement with literature data evaluated in acidic chloride solutions.^[^ ^10^ ^]^ Compared with acidic chloride solutions, the *N*_D_ values found at *E* > 1.5 V in neutral solutions are lower by almost a magnitude of order.

### 2.4 Breakdown of the Passivity on Nb and Transition to Stable Pit Active Dissolution

**Figure 3:**
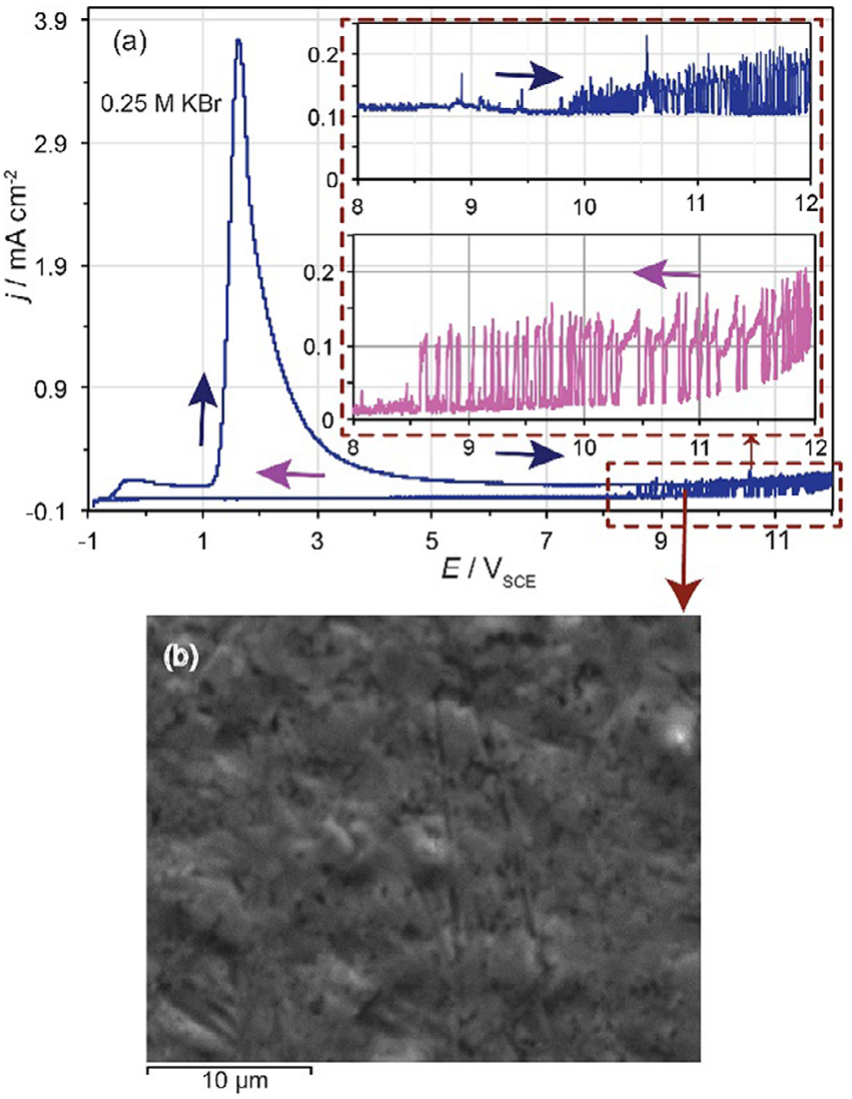
(a) Current density‐potential (*j*‐*E*) potentiodynamic curve of Nb traced at *υ* = 10 mV s^−1^ in 0.25 M KBr in the potential region −0.9 – 12 V. (b) SEM image of the passive Nb surface taken at the upper potential limit of 12 V. Insets in (a): enlargement of the potential region where current fluctuations appear during the forward and reverse backward potential scans indicated by arrows.

As mentioned above, the destructive effect of bromides on the passive state of Nb becomes noticeable in the absence of inhibiting anions. It may appear either as metastable or stable localized corrosion depending primarily on the bromide concentration and the rate of oxide formation. Figure 3a shows that the *j*–*E* curve of Nb traced at *υ* = 10 mV s^−1^ in 0.25 M KBr exhibits current fluctuations in the region between 6 and 12 V signifying metastable localized corrosion which cannot be stabilized at least up to 12 V. It seems that passivation is not completely prevented and local areas become temporarily active. However, a persistent increase of the anodic current beyond a critical potential value is not observed in 0.25 M KBr as in the case of 0.5 M KBr (Figure 1b).

Metastable pitting is characterized by a transient sudden increase of the current above the background passive current followed by a decayas can be seen in Figure 3a (insets). The observed current fluctuations comprise successive active‐passive events that manifest themselves in both the forward and reverse backward scans. Current fluctuations appeared for all potential scan rates used in this study, between 5 and 250 mV s^−1^. They are intensified upon decreasing the potential scan rate.

Successive activation‐passivation events associated with metastable pitting corrosion are considered as an intermediate stage prior to either the re‐passivation of the newly generated pits or the growth and stabilization of these pits.^[^ ^21^, ^28^ ^]^ The stabilization of pits is characterized by sustained dissolution of the exposed metal surface beyond the critical breakdown potential, *E*_b_ (Figure 1b). The metastable pitting in the case of Nb consists of pit‐nucleation sites in which, immediately after the passivity breakdown, the exposed underlying metal reacts for a while with the electrolyte and in turn, an immediate restoration of the Nb_2_O_5_ layer occurs. Restoration seems to prevail under the present conditions as repassivation of pits occurs rapidly due to the high tendency of Nb to form the oxide layer. Visual observation by an optical microscope as well as scanning electron microscope (SEM) images taken at 12 V (Figure 3b) show dark spots suggesting the film damage induced by bromides at localized surface sites. However, pit growth and its stabilization associated with a sustained metal dissolution appear only at higher bromide concentrations, beyond the *E*_b_, as depicted in Figure 1b at 0.5 M KBr. It will be shown below that same phenomena occur also for 0.75 and 1 M KBr. The dependence of the critical *E*_b_ on the bromide activity, *α*_Br_ and the potential scan rate, *υ* were examined as the effect of both *α*_Br_ and *υ* is anticipated to provide an insight into the mechanism underlying the passivity breakdown on Nb.^[^ ^16^, ^17^ ^]^

### 2.5 Effect of the Bromide Concentration on the Breakdown Potential

**Figure 4:**
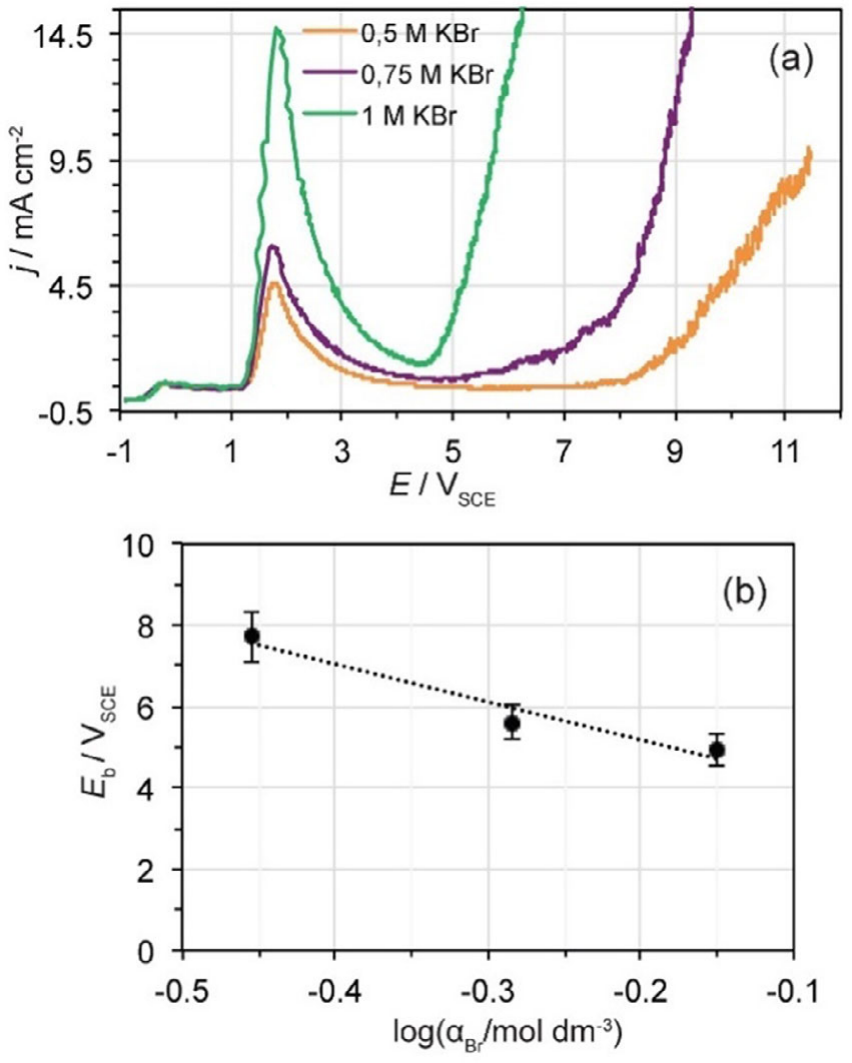
(a) Current density‐potential (*j‐*‐*E*) potentiodynamic curves of Nb traced at *υ* = 50 mV s^−1^ in KBr of different concentrations. (b) Effect of the bromide activity on the breakdown potential, *E*_b_ of the passive state on Nb.

Figure 4a shows the *j*‐*E* potentiodynamic curves of Nb traced in 0.5, 0.75, and 1.0 M KBr solutions at *υ* = 50 mV s^−1^. The ionic activity coefficient *γ*_Br_ for Br^−^ in binary KBr solutions of different concentrations was utilized to calculate the bromide activity, *α*_Br_ in the solutions.^[^ ^29^ ^]^ As can be seen in Figure 4b, the *E*_b_ decreases linearly with log*α*_Br_ for a constant *υ* in agreement with observations reported for the localized corrosion of many metals and alloys.^[^ ^30^ ^]^ The relationship *E*_b_ = f(log*α*_Br_) obtained from the *j*–*E* curves shown in Figure 4b is:

(3) $$E_{b}=3.313\left(\pm 0.435\right)-9.328\left(\pm 0.510\right)\log\alpha_{\mathrm{Br}}$$

Which can be compared with the relationship:

(4) $$\;E_{b}=E_{b}^{o}\;-\frac{2.303RT}{aF}loga_{X}$$

that is predicted by the PDM for pitting corrosion induced by halides X^−^.^[^ ^17b^ ^]^ In Equation (4), *α* is the polarizability of the Nb_2_O_5_|electrolyte interface which is estimated to be ∼0.006. The low value of *α* suggests that most of the applied potential emerges predominantly as a potential drop within the passive oxide film, in agreement with findings for other valve metals such as Ti.^[^ ^16^, ^26^ ^]^ It should be noted that Nb_2_O_5_ appears to be less susceptible to the bromide attack than TiO_2_ for which breakdown occurs at lower bromide concentrations and lower *E*_b_.^[^ ^16b^ ^]^

**Figure 5:**
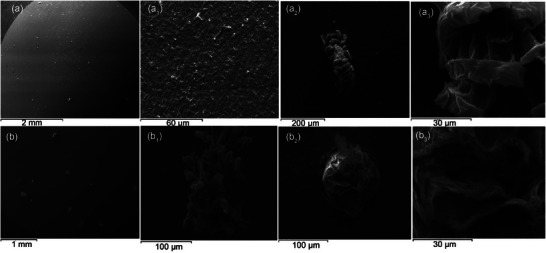
SEM micrographs of Nb surfaces taken at *E* > *E*_b_ for different values of the rising current density after the passivity breakdown and the establishment of stable pitting observed during a potential scan at *υ* = 10 mV s^−1^ in 0.75 M KBr. (a), (a_1_–a_3_) *j* = 1 mA cm^−2^ and (b), (b_1_–b_3_) *j* = 3 mA cm^−2^.

The higher breakdown resistance of passive Nb is also reflected in the form of localized corrosion occurring on Nb as compared with Ti. In the latter case, a relatively large area of the oxide passive film was completely removed whereas isolated hemispherical pits found to grow at *E* > *E*_b_ on the passive Nb surface, as can be seen in the SEM micrographs of Figure 5.

SEM images (a) and (b) of Figure 5 depict the overall surface for different values of the rising current density corresponding to *j* = 1 mA cm^−2^ and *j* = 3 mA cm^−2^, respectively. Different values of the rising current density represent different time moments and hence pit evolution. Comparing SEM images (a) and (b) shows that the population of pits nucleated at localized sites of the passive surface increases with time as expected for pitting corrosion, which is a time‐dependent process.^[^ ^31^ ^]^

As can be seen in Figure 5a_1_ tiny pits, nucleated at early stages are repeatedly re‐passivated by newly formed oxide though breakdown occurs only in few sites. Inspecting SEM images in Figure 5a_2_ and 5b_1_ shows that the growth of pits does proceed in few sites leading to various morphologies depending on the time at which images were taken. Even at these susceptible sites there is a continuous breakdown and repassivation as their morphology shows. Figure 5b_2_ illustrates an example of a deep hemispherical pit possessing a multilayered porous cover on top as can be seen in Figure 5b_3_, which displays the damaged area in a greater magnification. The composition of pit top‐covers examined by EDX found to comprise Nb and oxygen which were also found to prevail over the whole Nb disc electrode surface.

### 2.6 Effect of the Potential Scan Rate on the Breakdown Potential

**Figure 6:**
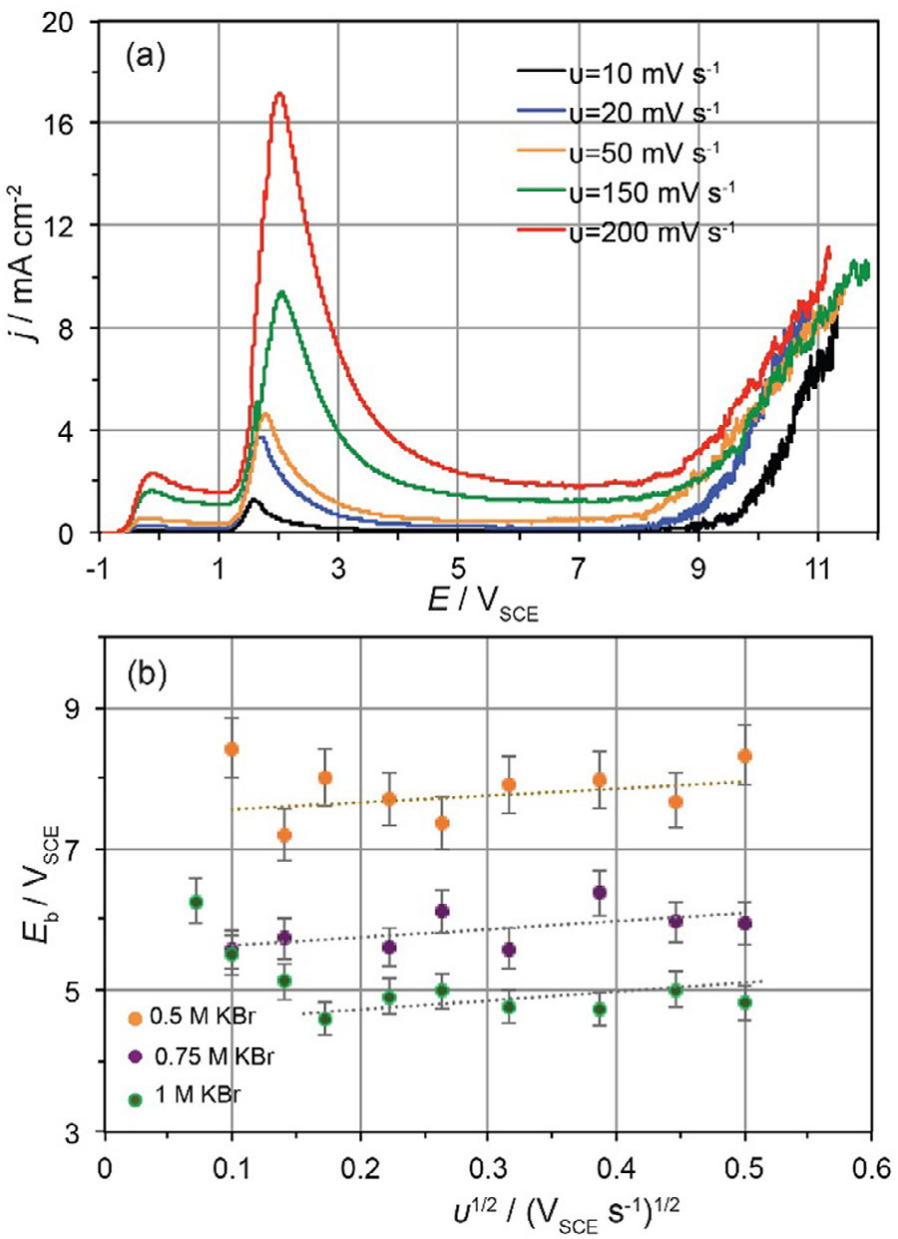
(a) Potentiodynamic current density‐potential (*j*‐*E*) curves of Nb traced at different potential scan rates, *υ* in 0.5 M KBr. (b) Dependence of the breakdown potential, *E*_b_ on *υ* ^1/2^.

To get an insight into the competing processes comprising the oxide growth and the bromide‐induced breakdown of the passivity on Nb, the effect of the potential scan rate (*υ*) on *E*_b_ was also studied. Measurements at different *υ* ranged between 10 and 250 mV s^−1^ were carried out for all bromide concentrations used in this study. An example of the effect of *υ* on the anodic *j*‐*E* potentiodynamic curves of Nb in 0.5 M KBr is shown in Figure 6 for selected values of the potential scan rate.

Upon increasing *υ*, there is a relatively lesser effect in comparison with that observed in the case of passive Ti^[^ ^16b^ ^]^ and other metal and alloys in neutral media.^[^ ^24b^ ^]^ Nevertheless, the mean values of *E*_b_ obtained from 5–6 independent experiments exhibit a trend for a linear increase on *υ* ^1/2^ (Figure 6b) for *υ* > 50 mV s^−1^ with a slope that is rather constant at various bromide concentrations. It seems from Figure 6b that as the bromide concentration increases this tendency is not clear enough in the range of 5 – 50 mV s^−1^. Within the latter potential scan region, the *E*_b_ exhibits a rather decreasing tendency with *υ* ^1/2^, which is a reverse dependency than the expected one.

Localized corrosion is a time‐dependent process and, the longer the polarization time (lower *υ*) the greater the extent the localized corrosion is expected to evolve. M–S analysis reveal that for films formed at *υ* < 50 mV s^−1^, the donor density, *N*_D_ increases by decreasing *υ* as the *E*_b_ does (Figure S4). This might be rationalized by considering the greater crystallinity and hence conductivity^[^ ^1^ ^]^ of these films along with their higher thickness. Though there is time for the bromide action the higher thickness prevents breakdown shifting the *E*_b_ to higher values. At *υ* > 50 mV s^−1^, the *N*_D_ increases upon increasing *υ* (Figure S4) since the films formed at faster rates of growth are in general thinner with different structural features. In this case, the time for bromide action is restricted and the expected dependence of *E*_b_ on *υ* ^1/2^ is observed. The *E*_b_ increases with *υ* indicating that the localized breakdown is prevented at higher *υ*.

The relationship *E*_b_ = f(*υ* ^1/2^) obtained from the plots of Figure 6b at *υ* > 50 mV s^−1^ is as follows:

(5) $$E_{b}=\varepsilon_{b}\left(v=0\right)+0.94\left(\pm 0.05\right)v^{1/2}$$

where the *E*_b_(*υ* = 0) is the breakdown potential under steady state conditions (*υ* = 0), which decreases upon increasing the bromide concentration. The *E*_b_(*υ* = 0) values being approximately 7.7 V_SCE_ (0.5 M KBr), 5.5 V_SCE_ (0.75 M KBr), and 4.9 V_SCE_ (1 M KBr) are close to those found at *υ* = 50 mV s^−1^ (Figure 4b). According to the PDM, the relationship *E*_b_ = f(*υ* ^1/2^) is:

(6) $$\;E_{b}=E_{b}\;\left(v=0\right)+{\left(\frac{2\xi RT}{J_{m}\chi Fa}\right)}^{1/2}v^{1/2}$$

where *ξ* is the critical arial concentration (mol cm^−2^) of condensed cation vacancies at the Nb|Nb_2_O_5_ interface necessary for the breakdown of the oxide, *J*_m_ is the flux by which cation vacancies are adsorbed into the metal, *χ* is the charge of metal in the oxide lattice, and *α* is the polarizability of the Nb_2_O_5_|electrolyte interface calculated above by comparing Equations (3) and (4).

## 3 Discussion

The experimental findings of this study show that in both neutral and acidic aqueous media, the active‐passive transition of Nb occurs at relatively low potentials and the passive oxide film exhibits excellent stability in acid and chloride‐containing media. This might be assigned to the spontaneous formation of the oxide (*ΔG* ^ο^ = −1853.1 kJ mol^−1^ for Νb_2_O_5_) and its high resistance found in most aqueous solutions including chloride‐containing ones (Figure 1a). Previous studies show that in the case of Ti (*ΔG* ^ο^ = ‐888.8 kJ mol^−1^ for the formation of TiO_2_) chlorides does induce metastable pitting corrosion.^[^ ^24a^ ^]^ Surprisingly, the less aggressive bromides cause stable pitting corrosion of the passivity on Ti.^[^ ^14^, ^16^ ^]^ As was shown above, this is also the case for the passivity on Nb (Figure 1b), which exhibits a relatively high susceptibility to localized corrosion in neutral bromide aqueous solutions. This agrees with previous observations.^[^ ^13^ ^]^ However, the experimental relationships *E*_b_ = f(log*α*_Br_) and *E*_b_ = f(*υ* ^1/2^) for the breakdown potential of the passivity on Nb in bromide‐containing solutions are evaluated for the first time in the present study in accord with the predictions of the PDM.

**Scheme 2:**
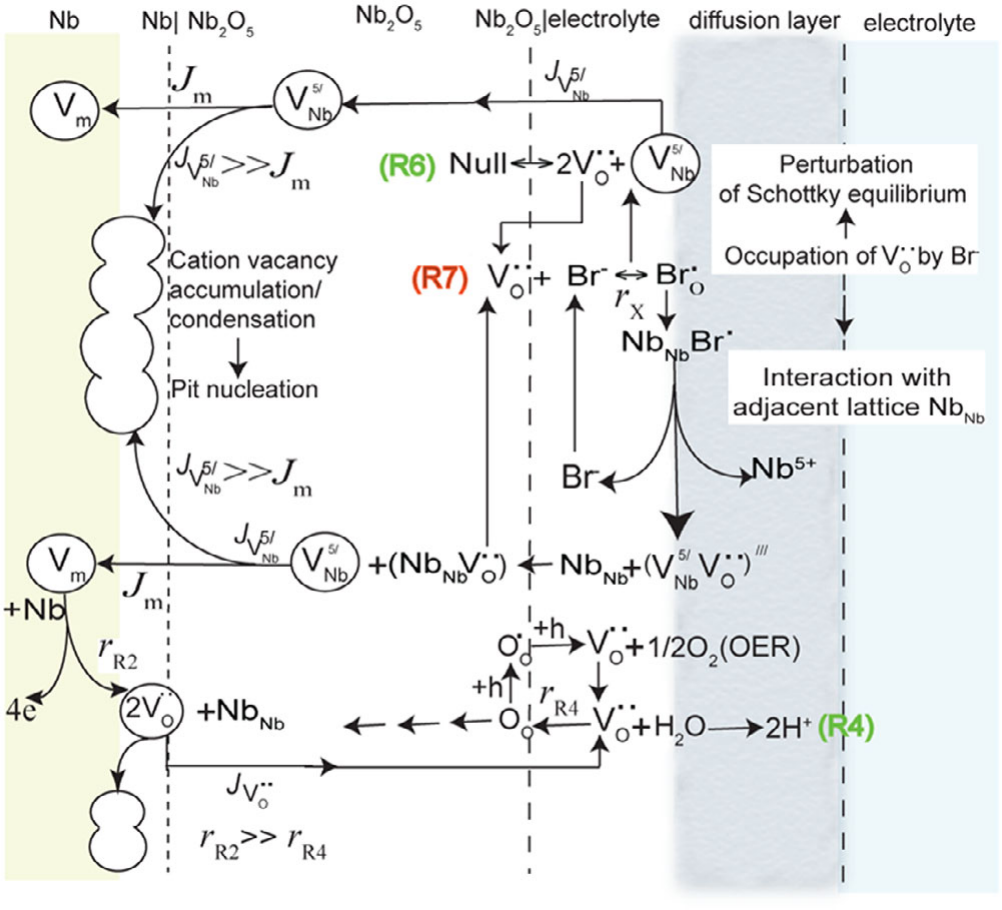
Schematic representation of physicochemical processes that presumably lead to the accumulation of either cation or anion vacancies at the Nb|Nb_2_O_5_ interface that initiate pit nucleation.

Though the PDM has previously been used by several authors^[^ ^22^, ^23^ ^]^ to describe the oxide growth on Nb, the breakdown of the passivity on Nb has not yet been described in terms of the PDM. Reaction (R7) included in Scheme 1, should be considered to occur, after bromide dehydration (Br^−.^nH_2_O ↔ Br^−^ + nH_2_O) in competition with reaction (R4) at the Nb_2_O_5_|electrolyte interface. It implies the occupation of oxygen vacancies by dehydrated Br^−^ instead of O^2−^ resulting in the generation of new oxygen vacancies for the electroneutrality to be kept according to the Schottky equilibrium, reaction (R6). The resulting $Br_{O}^{.}$ species is a bromide ion that occupying an oxygen vacancy in the oxide lattice can be represented by $Nb_{Nb}Br_{O}^{.}$ as shown in Scheme 2. The extraction of the Nb_Nb_ lattice cation may occur at the Nb_2_O_5_|electrolyte interface. This results in the generation of a surface cation vacancy/anion vacancy pair and regeneration of Br^−^ at the Nb_2_O_5_|electrolyte interface. Anion vacancies might be reoccupied by either O^2−^ (passivating species) or Br^−^ (breakdown‐activating species) whereas cation vacancies are transferred across the oxide layer towards the Nb|Nb_2_O_5_ interface by a flux $J_{V_{\mathrm{Nb}}^{5^{\prime }}}$.

Apparently, the generation of cation vacancies is an autocatalytic process and reaction (R7) is a key reaction for subsequent processes to occur resulting in the accumulation of cation vacancies at the Nb|Nb_2_O_5_ interface as the flux of transport of cation vacancies becomes much higher than the flux of the annihilation of cation vacancies at the Nb|Nb_2_O_5_ interface ($J_{V_{\mathrm{Nb}}^{5^{\prime }}}>$ *J*_m_) under steady state conditions. Based on Equation (6), the critical arial concentration *ξ* of condensed cation vacancies at the Nb|Nb_2_O_5_ interface can be estimated using the slope of the *E*_b_ = f(*υ* ^1/2^) line, Equation (5), as the efficient charge of the cation vacancy, *χ* is known, and *α* was found by comparing Equations (3) and (4) to be equal to 0.006. The flux *J*_m_ of cation vacancies annihilated at the Nb|Nb_2_O_5_ interface should be at least equal to the flux $J_{V_{\mathrm{Nb}}^{5^{\prime }}}$ of cation vacancies across the oxide layer for the vacancy condensation to be avoided and can be estimated by using the passive current density, *j*_pas_ prior the oxide breakdown *J*_m_ =$J_{V_{\mathrm{Nb}}^{5^{\prime }}}\;\leq$ *j*_pas_ *N*/*χF*, where *N* is the Avogadro constant (6.022 × 10^23^) and *χ* = 5. The inequality in the abovementioned relationship stands to account for the fact that the niobium oxide exhibits *n*‐type semiconductive properties and hence the dominant defects are not the cation vacancies initiating the breakdown of the passivity but the oxygen vacancies. Therefore, the current density due to the flux $J_{V_{\mathrm{Nb}}^{5^{\prime }}}$ should be less than that corresponding to the passive current density *j*_pas_ determined by oxygen vacancies due to the $J_{V_{\ddot{O}}}$. For current density *j*_pas_ = 0.5 mA cm^−2^ the flux *J*_m_ is evaluated to be less than 6.24 × 10^14^ cm^−2^ s^−1^. In turn, based on Equations (5) and (6), the critical concentration of condensed cation vacancies is found to be *ξ* < 3.22 × 10^14^ cm^−2^. This value is relatively lower compared with that found for passive Ti^[^ ^16b^ ^]^ and comparable with ξ values found for stainless steel of different types, either AISI 403^[^ ^24b^ ^]^ or AISI 316L.^[^ ^32^ ^]^ It should be noted that, as in the case of AISI316L, either the condensation of oxygen vacancies (Scheme 2) or the association of cation/anion vacancy occurring when cation vacancies cannot be annihilated instantaneously at the Nb|Nb_2_O_5_ interface might happen. Both are possible to be involved in the case of the passivity breakdown on Nb. This is further supported by the high density of oxygen vacancies (10^19^–10^21^ cm^−3^) found by using the M–S analysis (Table 1). Association of cation vacancies may grow and lead to the formation of a void as the flux $J_{V_{\mathrm{Nb}}^{5^{\prime }}}$ becomes progressively higher than the *J*_m_ (Scheme 2) due to the continuous generation of cation vacancies initiated by reaction (R7).^[^ ^24^, ^33^ ^]^ On the other hand, association of anion vacancies at the Nb|Nb_2_O_5_ interface is also possible when the generation rate of oxygen vacancies, *r*_R2_ is accelerated with potential and by the oxygen evolution reaction (OER), which results in a reduced rate, *r*_R4_ of the injection of O_O_ toward the Nb|Nb_2_O_5_ interface. The oxide breakdown at voids is facilitated by a simultaneous thinning of the oxide film via a bromide‐assisted general dissolution reaction.

The higher aggressiveness of bromides relative to chlorides can be interpreted by considering the electrochemical activity of bromides on the Nb|Nb_2_O_5_ electrode facilitating their adsorption and occupation of oxygen vacancies across the oxide|electrolyte interface in competition with O^2−^, as in the case of the passivity breakdown on Ti.^[^ ^16b^ ^]^ This is also in agreement with previous results obtained for the Nb|Nb_2_O_5_ electrode with the use of tracer Br^82^ in KBr solutions.^[^ ^13^ ^]^ It was observed that the surface concentration of Br^−^ starts to increase at potentials where bromide electrochemical oxidation occurs and keeps increasing upon increasing further the applied potential.

## 4 Conclusion

This work shows that the active‐passive transition of Nb in halide‐containing neutral media occurs at low potentials, close to the open circuit potential of the Nb|electrolyte interface as it does in acid and alkaline media. The Nb anodic oxide film formed in sulfuric acid solutions as well as in chloride‐ and bromide‐containing sulfuric acid solutions is stable in contrast to single bromide solutions. Localized breakdown occurs by bromides which unexpectedly appear to be more aggressive than chlorides. Passive Nb exhibits stable active dissolution occurring at a high dissolution rate in pits generated at potential values higher than the critical breakdown potential, *E*_b_ determined by using potentiodynamic *j*‐*E* curves.

The *E*_b_ found for the passivity breakdown on Nb depends on both the bromide concentration and potential scan rate in agreement with the predictions of the PDM. Slight deviations are observed at potential scan rates lower than 50 mV s^−1^, an effect that can presumably be assigned to structural changes of the oxide film grew on Nb at low scan rates characterized by higher thickness and crystallinity as compared with that formed rapidly at higher scan rates. A competitive adsorption of oxygen ions (passivator) and bromides (activator) on oxygen vacancies occurs constantly and thus promotion of either the oxide growth or oxide breakdown depends on the film structure, the bromide concentration, and the potential scan rate at constant temperature and solution pH.

Using the M–S analysis shows that bromides result in an increase of the donor density in the oxide layer and hence of the oxygen vacancies that are considered as the predominant oxide defects. Vacancy condensation is considered to occur across the Nb|Nb_2_O_5_ interface leading to the local formation of a void and hence to decohesion of the oxide from the substrate. This prevents the oxide growth resulting in susceptible to breakdown local sites. The estimated critical arial vacancy concentration for the local oxide breakdown to occur is in fair agreement with values estimated for other metals and alloys.

## 5 Experimental Section

### Materials and Equipment

Niobium rod (99.9% pure) with a diameter equal to 5 mm was supplied by Goodfellow Cambridge Limited. Working electrodes were made by embedding rod specimens in a 1 cm diameter PTFE cylinder. The exposed surface (5 mm in diameter, surface area 0.196 cm^2^) was polished by a series of wet‐sand papers of different grit sizes (500, 800, 1000, 1200, and superfine). After the mechanical polishing, the electrode was cleaned in an ultrasonic bath containing twice‐distilled water.

A three‐electrode cell connected to a VoltaLab 40 electrochemical system and controlled by the VoltaMaster 4 software from radiometer analytical was used. A Pt sheet (2.5 cm^2^) and a saturated calomel electrode (SCE) were served as counter and reference electrodes, respectively. Potential values are reported with respect to the SCE electrode. Electrolyte solutions were prepared using H_2_SO_4_ (Merck, pro‐analysis 96% w/w), Na_2_SO_4_ (Fluka, puriss p.a.), KCl (Fluka, puriss p.a.), KBr (Fluka, puriss p.a.) and, twice‐distilled water. A volume of 150 mL was maintained in a three‐electrode electrochemical cell. All measurements were carried out at ∼293 K with N_2_ deaeration.

## Methods

Cyclic and linear sweep voltammetry were conducted within the potential region from −0.9 to 12 V_SCE_ to characterize the electrochemical behavior of the Nb|Nb_2_O_5_ interface in various aqueous media. The breakdown potential *E*_b_ induced by Br^−^ was determined in bromide‐containing solutions using current density‐potential (*j*‐*E*) curves traced at different potential scan rates, *υ* ranged from 10 to 250 mV s^−1^. The identification of *E*_b_ was based on a critical current density equal to 1 mA/cm^2^ estimated from the base line for each *j*‐*E* curve. To justify the effect of either the bromide activity in solution, *α*_Br_ or of *υ* on *E*_b_, 5–6 measurements were performed for each specimen. Localized corrosion of the Nb electrode surface was visualized via an optical microscope whereas the surface morphology was examined using a JEOL JSM‐840A scanning electron microscope (SEM).

Capacitance measurements for the M–S analysis were performed at a frequency of 5 kHz by polarizing the Nb|Nb_2_O_5_ interface at a selected potential reached by scanning at the same *υ* used for the determination of *E*_b_. A sinusoidal potential perturbation of 10 mV in amplitude was applied at the selected potential. Polarization started at this potential and ended at −0.7 V with an increment equal to 0.05 V.

## Conflict of Interests

The authors declare no conflict of interest.

## Supporting information



Supporting Information

## Data Availability

The data that support the findings of this study are available from the corresponding author upon reasonable request.
