# Supplementary material for: Localized Breakdown of the Niobium Anodic Oxide Film by Bromides
Source: Chemistry. 2025 May 3;31(32):e202500398. doi: 10.1002/chem.202500398 (PMC12144905; doi:10.1002/chem.202500398)
Supplement: Supplementary file 1 — Supporting Information [file CHEM-31-e202500398-s001.docx]

Supporting Information

Localized Breakdown of the Niobium Anodic Oxide Film by Bromides

Eirini Lappa, Kyriaki Saltidou, Chrysanthi Gkili and Dimitra Sazou*

Department of Chemistry, Aristotle University of Thessaloniki, 54124 Thessaloniki, Greece

*Corresponding author.

E-mail: [sazou@chem.auth.gr](mailto:sazou@chem.auth.gr)

**The effect of various anions on the electrochemical behavior of Nb**


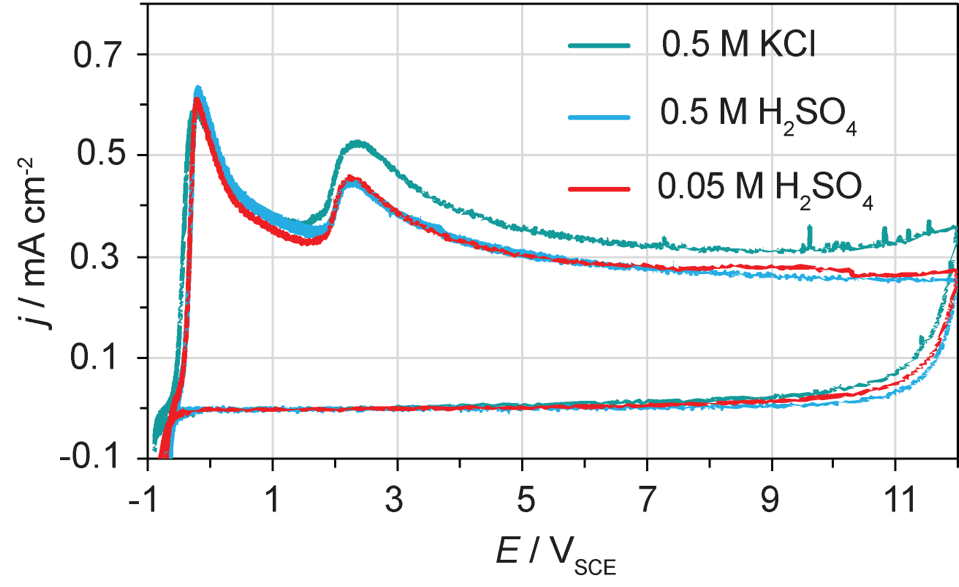


**Figure S1**. Current density-potential (*j*-*E*) potentiodynamic curves of Nb traced at *υ* = 50 mV s^-1^ within the region -0.9 V - 12 V in 0.5 M KCl, 0.5 M H_2_SO_4_ and 0.05 M H_2_SO_4_. Current density of peak **a_2_** is slightly higher in KCl than in sulfuric acid solutions indicating that chlorine evolution may also occur along with oxygen evolution. However, the corresponding peak current density in 0.5 M KBr (Figure 1b) is approximately 20 times higher than in 0.5 M KCl. This show that electrochemical oxidation of chlorides may occur but to a much-lowered extent than that of bromides.

**
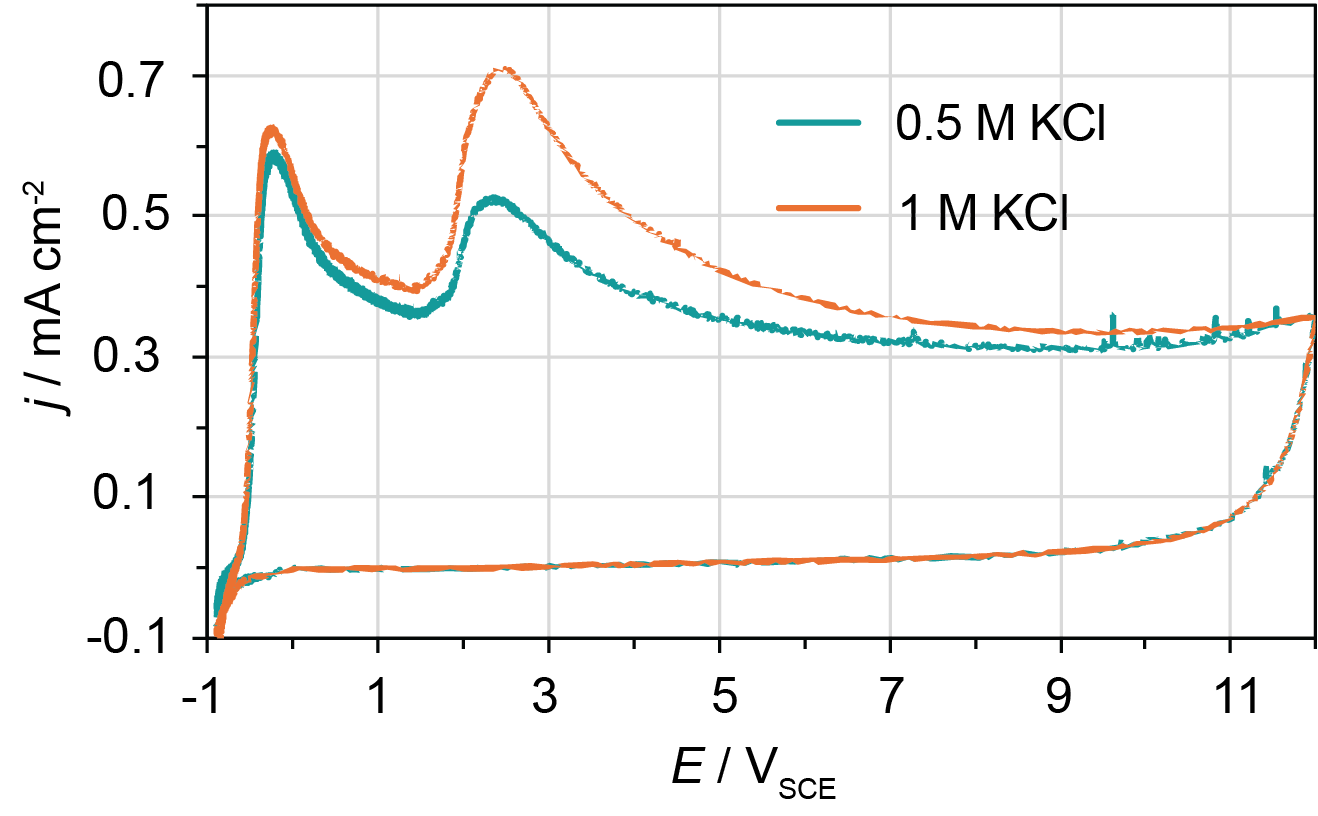
**

**Figure S2**. Comparing the current density-potential (*j*-*E*) potentiodynamic curves of Nb traced at *υ* = 50 mV s^-1^ within the region -0.9 V - 12 V in 0.5 and 1 M KCl shows that the current density of peak **a_2_** increases upon increasing the chloride concentration. This seems to support the occurrence of chlorine evolution at peak **a_2_** along with oxygen evolution. Apparently, the rate of this electrochemical reaction is much lower than the rate of the corresponding bromide electrochemical oxidation as the current density of peak **a_2_** in 1 M KBr (Figure 4a) is approximately 45 times higher than in 1 M KCl.

**
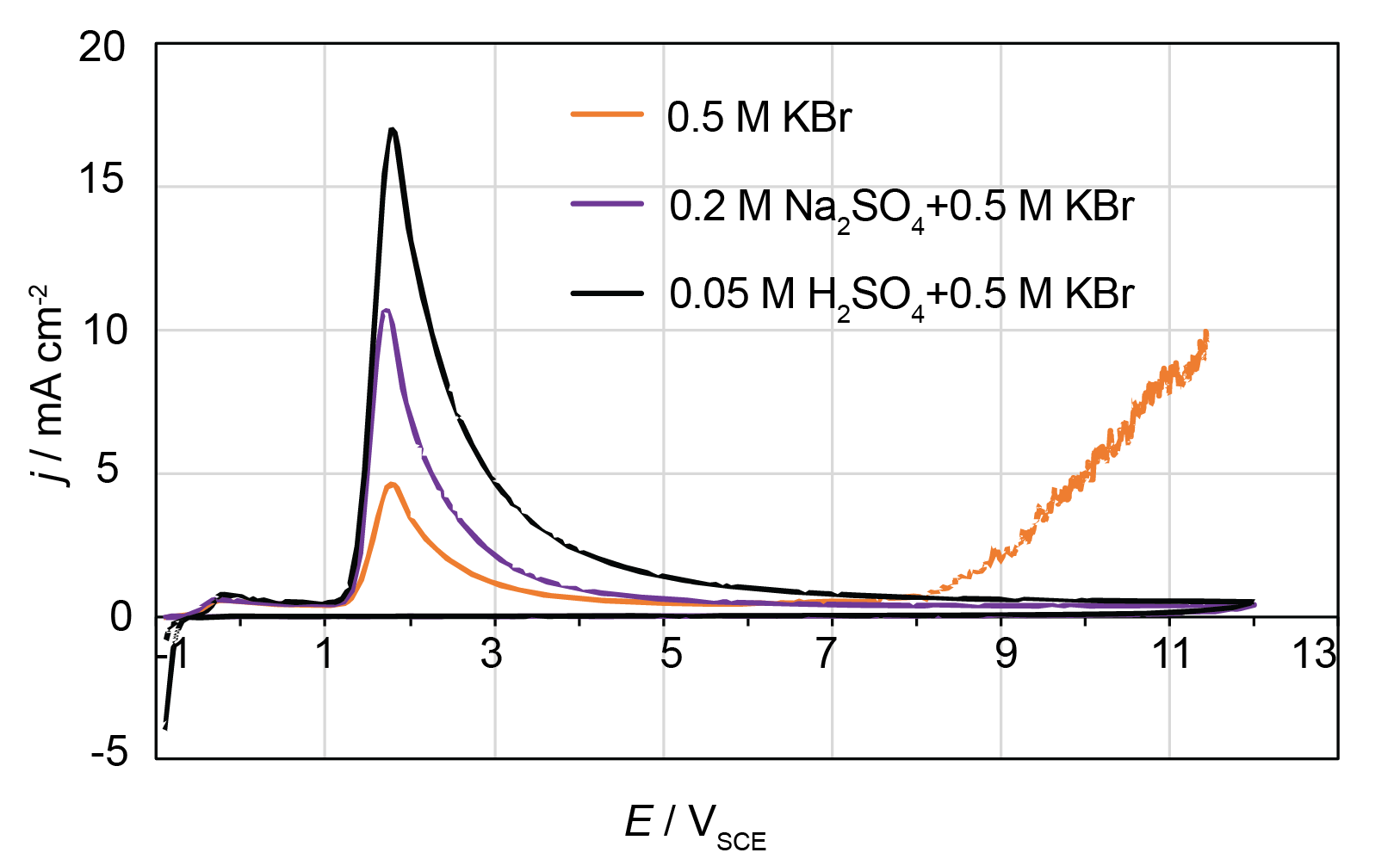
**

**Figure S3**. Current density-potential (*j*-*E*) potentiodynamic curves of Nb traced at *υ* = 50 mV s^-1^ within the region -0.9 V – 12 V in 0.5 M KBr in the absence and presence of either 0.05 M H_2_SO_4_ or 0.2 M Na_2_SO_4_. The obtained *j*-*E* curves indicate that sulfates promote the bromide electrochemical oxidation.

**The effect of the potential scan rate on the oxide growth and its bromide-induced breakdown**

Figure S4 shows a correlation between the breakdown potential, *E*_b_ and the donor density, *N*_D_ estimated by using the analysis of Mott-Schottky (M-S) curves recorded for oxide passive films formed on Nb by scanning the potential region -0.9 V - 6 V in 0.5 M KBr at different scan rates, *υ*. This correlation is indicative of the competition between different processes associated with the oxide growth and its breakdown.

In terms of the point defect model (PDM), *E*_b_ increases linearly with *υ*^1/2^ and this is indeed the case for potential scan rates, *υ* > 50 mV s^-1^ as is expected. Upon increasing *υ*, the time for bromide action is restricted and thus localized breakdown is prevented. Accordingly, the *N*_D_ increases upon increasing *υ* since the films formed at faster rates of growth are in general thinner with different structural features.

On the other hand, at potential scan rates lower than 50 mV s^-1^, both *N*_D_ and *E*_b_ increase by decreasing *υ*. This can perhaps be rationalized by considering structural changes in the film because at low rates of growth the crystallinity of oxide films is often favored and in turn the conductivity increases. On the other hand, at relatively low *υ*, the thickness of the film increases and though there exist time for the bromide action and the accumulation of cation vacancies across the Nb|Nb_2_O_5_ interface, as is suggested by the PDM (Scheme 2), local thinning of the surface film via a general dissolution requires a longer time. By increasing *υ* at 20 mV s^-1^ and then at 50 mV s^-1^ the latter process is facilitated as the film is thinner and breakdown occurs at lower *E*_b_.

**
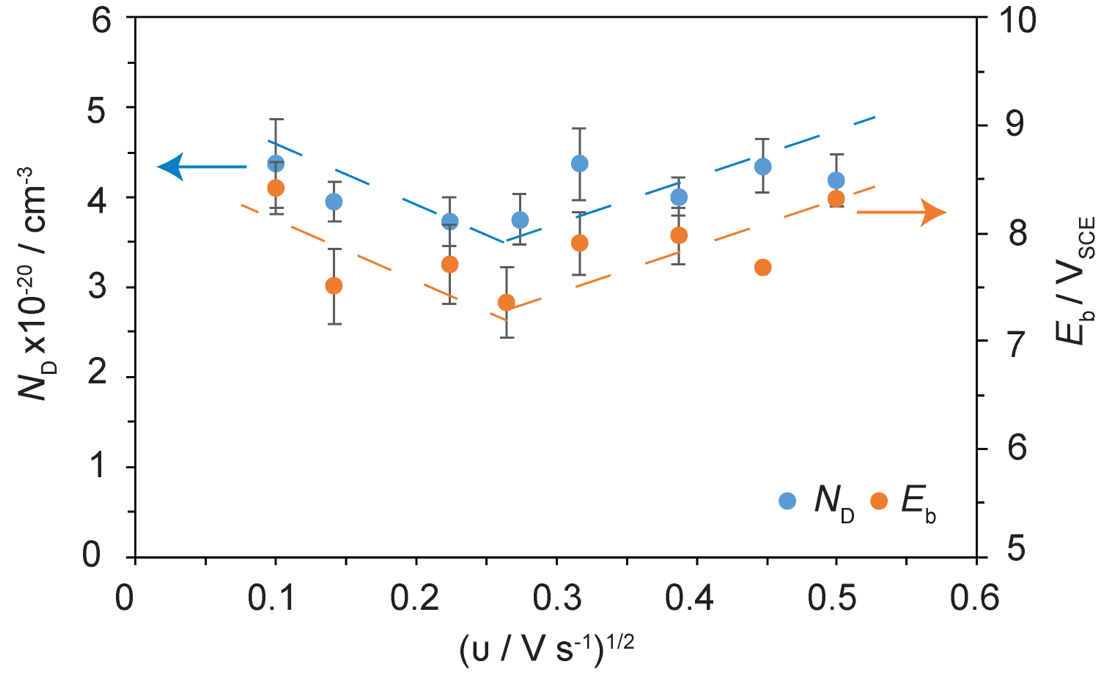
**

**Figure S4**. Correlation between the breakdown potential, *E*_b_ and the donor density, *N*_D_ estimated by using Mott-Schottky (M-S) curves recorded for oxide passive films formed on Nb by scanning the potential region between -0.9 V and 6 V in 0.5 M KBr at different scan rates, *υ*. Selected conditions for M-S curves resemble those used for the identification of *E*_b_. *E*_b_ and *N*_D_ data points are plotted vs *υ*^1/2^ while the dashed lines in the figure are guide for the eye. Two different types of behavior are observed indicating a competition between the processes leading to the growth of the oxide and the bromide-based processes that induced its localized breakdown.
